# Coinciding Placental Abruption and Pulmonary Edema in a Patient With Preeclampsia

**DOI:** 10.7759/cureus.66065

**Published:** 2024-08-03

**Authors:** Shannon Williams, Ciauna Tran, Shanon Forseter

**Affiliations:** 1 Obstetrics and Gynecology, Rocky Vista University College of Osteopathic Medicine, Englewood, USA; 2 Obstetrics and Gynecology, North Suburban Medical Center, Thornton, USA

**Keywords:** cesarean section, placental abruption, postpartum pulmonary edema, postpartum dyspnea, preeclampsia with severe features

## Abstract

Preeclampsia is a known complication of pregnancy. Patients meet diagnostic criteria when they have hypertension along with proteinuria and/or end-organ dysfunction. Preeclampsia can pose a serious threat to the lives of pregnant patients and their fetuses. A 35-year-old G4P0030 female was diagnosed with preeclampsia at 35 weeks gestation. She was scheduled for an induction of labor at 37 weeks. With further elevation in blood pressure during labor, she met the criteria for preeclampsia with severe features. Additionally, the presence of clinical signs concerning a placental abruption led to a cesarean delivery. Following her delivery, her respiratory distress prompted a computed tomography angiography, which showed evidence of pulmonary edema. The occurrence of both placental abruption and pulmonary edema can be related to the patient’s preeclampsia. We urge that healthcare providers have a low threshold for diagnosing placental abruption and pulmonary edema in patients with preeclampsia.

## Introduction

Preeclampsia is a hypertensive disorder of pregnancy that is characterized by proteinuria and/or end-organ dysfunction [1,2]. The condition is thought to be an outcome of abnormal placentation wherein embryo trophoblasts fail to penetrate into the spiral arteries and transform into endothelial phenotype. This results in placental ischemia and the release of angiogenic factors and oxidative stress that causes diffuse endothelial dysfunction in the pregnant patient’s vasculature [1]. Estimates show that preeclampsia occurs in 4.6% of all deliveries worldwide [2]. Preeclampsia can seriously complicate a patient’s pregnancy as it is associated with life-threatening conditions. The disorder has been identified as a risk factor for placental abruption, which only occurs in <1% of all pregnancies but can lead to negative outcomes for both pregnant patients and their fetuses [3]. Additionally, pulmonary edema is a severe feature of preeclampsia. Despite occurring in only 0.08% of pregnancies, pulmonary edema is the leading cause of maternal mortality [4,5]. The following case report gives us the opportunity to discuss multiple uncommon but severe complications of preeclampsia in a patient who underwent a cesarean delivery for placental abruption and then subsequently developed pulmonary edema in the postpartum period. As will be discussed, these complications necessitate early recognition and prompt management for the safety of patients and their fetuses.

## Case presentation

A 35-year-old G4P0030 female, with an unremarkable past medical history, presented to her initial obstetrics visit at 16 weeks. She was started on aspirin 81 mg daily due to advanced maternal age. At that time, she had an elevated blood pressure of 130/90 mmHg, but her home blood pressure was reportedly within normal limits. At her 30-week appointment, her blood pressure had increased to 142/93 mmHg. At 35 weeks, she had a similar blood pressure of 149/96 mmHg, which prompted measurement of a urinary protein/creatinine ratio that resulted in an elevated value of 0.53, hence meeting the criteria for preeclampsia. Her prenatal visits were increased to twice weekly, and she was scheduled for an induction of labor at 37 weeks. The patient's hospital course is summarized in Table 1.

**Table 1:** Timeline of hospital course POD, Post-operative day; SBP, Systolic blood pressure; CTA, Coronary computed tomography angiography; RSV, Respiratory syncytial virus

​	Admission/POD 0​	POD 1​	POD 2​	POD 3​
Signs/Symptoms​	SBP > 170 mmHg​ intermittent late decelerations​	Dyspnea and increasing oxygen requirements​ afebrile​	Increased urine output​ improvement of respiratory symptoms​ decreased oxygen requirements​	Adequate oxygen saturation on room air​
Labs/Imaging​	Leukocytosis​ elevated urine protein/creatinine ratio of 0.99,​ normal platelet counts and liver enzymes​	CTA shows evidence of pulmonary edema and pneumonia​ leukocytosis negative COVID-19, influenza, and RSV​	Echocardiogram shows pulmonary hypertension​	Increased leukocytosis​
Management​	Magnesium sulfate and labetalol​ Cesarean delivery​	Intravenous furosemide​, pulmonary hygiene and incentive spirometry​	Nebulizer treatment:, ceftriaxone and azithromycin​	Discharged with cefdinir, furosemide, and labetalol​

The patient was admitted to the labor and delivery unit at 37 weeks for her scheduled induction. Prenatal labs showed that she was O+ and negative for commonly tested infectious diseases, including group B Streptococcus in the vaginal swab. Labs on admission, as shown in Tables 2-3, were notable for urine protein/creatinine ratio of 0.99 and leukocytosis, as well as normal platelet counts and liver enzyme levels. A transabdominal ultrasound confirmed the fetus in cephalic positioning. Following a reactive non-stress test, induction of labor was initiated with misoprostol 50 mcg orally. While her cervix was 1 cm dilated, the fetal heart rate dropped to 90-100 bpm for six minutes but improved following maternal repositioning. An epidural was placed by anesthesia at this time, followed by a fetal scalp electrode and an intrauterine pressure catheter. Upon artificial rupture of membranes with the fetal scalp electrode, a significant flow of blood from the vagina was observed. The patient’s systolic blood pressure rose above 170 mmHg. At this point, magnesium sulfate and labetalol were administered. Electronic fetal monitoring revealed intermittent late decelerations, which persisted despite immediate patient repositioning.

**Table 2:** Hematology data POD, Post-operative day; WBC, White blood cell count; RBC, Red blood cell count; Hgb, Hemoglobin; Hct, Hematocrit; MCV, Mean corpuscular volume; MCH, Mean corpuscular hemoglobin; RDW-CV, Red cell distribution width-coefficient of variation; Plt, Platelet

​	Admission/POD 0​	Third Trimester Range [6]​	POD 1​	POD 3​	Nonpregnant Adult Range [6]​
WBC (x103/mm^3^)​	15.08​	5.6-14.8​	16.25​	17.80​	3.5-9.1​
RBC (x106/mm^3^)​	4.13​	2.71-4.43​	3.89​	3.35​	4.0-5.2​
Hgb (g/dL)​	9.6​	9.5-15.0​	9.1​	7.8​	12-15.8​
Hct (%)​	30.4​	28.0-40.0​	29.7​	25.3​	35.4-44.4​
MCV (µm^3^)​	73.6​	81-99​	76.3​	75.5​	79-93​
MCH (pg/cell)​	23.2​	29-32​	23.4​	23.3​	27-32​
RDW-CV (%)​	15.7​	12.7-15.3​	16.0​	16.4​	<14.5​
Plt (x109/L)​	397​	146-429​	388​	440​	165-415​
Neutrophils # (x103/mm^3^)​	11.42​	3.9-13.1​	12.83​	13.27​	1.4-4.6​
Lymphocytes # (x103/mm^3^)​	2.52​	1.0-3.6​	2.57​	2.99​	0.7-4.6​
Monocytes # (x103/mm^3^)​	0.66​	0.1-1.4​	0.52​	0.90​	0.1-0.7​
Eosinophils # (x103/mm^3^)​	0.15​	0-0.6​	0.080​	0.35​	0-0.6​
Basophils # (x103/mm^3^)​	0.04​	0-0.1​	0.05​	0.07​	0-0.2​

**Table 3:** Chemistry data POD, Post-operative day

​	Admission/POD 0​	Third Trimester Range [6]​	POD 1​	POD 3	Nonpregnant Adult Range [6]​
Urea nitrogen (mg/dL)​	7​	3-11​	8​	15​	7-20​
Creatinine (mg/dL)​	0.64​	0.4-0.9​	0.58​	0.67​	0.5-0.9​
Sodium (mEq/L)​	139​	130-148​	135​	141​	136-146​
Potassium (mEq/L)​	3.5​	3.3-5.1​	4.0​	3.2​	3.5-5​
Chloride (mEq/L)​	112​	97-109​	109​	112​	102-109​
Calcium, total (mg/dL)​	8.6​	8.2-9.7​	7.4​	7.8​	8.7-10.2​
Protein, total (g/dL)​	6.3​	5.6-6.7​	5.3​	​	6.7-8.6​
Albumin (g/dL)​	2.2​	2.3-4.2​	1.6​	​	4.1-5.3​
Bilirubin, total (mg/dL)​	0.2​	0.1-1.1​	0.3​	​	0.3-1.3​
Alkaline phosphatase (U/L)​	214​	38-229​	168​	​	33-96​
Aspartate transaminase (U/L)​	16​	4-32​	26​	​	12-38​
Alanine transaminase (U/L)​	19​	2-25​	18​	​	7-41​
Lactate dehydrogenase (U/L)​	208​	82-524​	​	​	115-221​
Uric acid (mg/dL)​	5.2​	3.1-6.3​	​	​	2.5-6.5​

The patient was quickly transported to the operating room for a low transverse cesarean section due to vaginal bleeding noticed upon rupture of membranes, elevated blood pressures, and non-reassuring fetal heart tracing. Bloody fluid was noted at the time of hysterotomy. The fetus was in vertex position and had a loose nuchal cord. Following delivery of the female infant, the placenta was easily removed from the uterus. The patient’s placenta was sent to pathology. Estimated blood loss was 600 mL, and quantitative blood loss was 260 mL. The infant weighed 2,381 g and Apgar scores at one minute and five minutes were 8 and 8, respectively. Both the mother and infant were noted to be stable following delivery.

**Figure 1:**
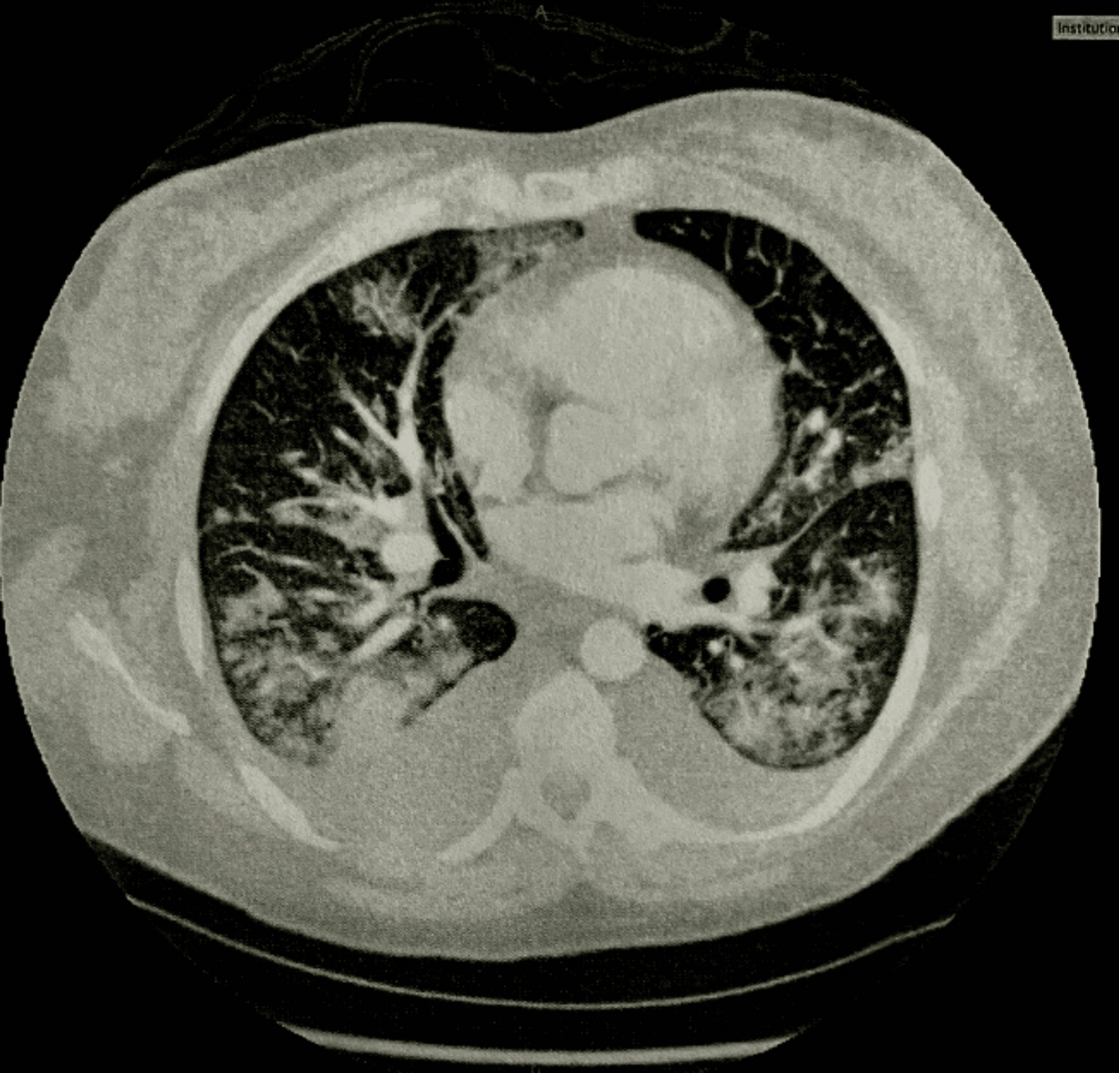
Computed tomography angiography (CTA) chest performed post-operative day one Impression reported pulmonary edema with bilateral pleural effusions, extensive groundglass and airspace disease, a developing consolidation in the right lower lobe, as well as a prominent central pulmonary artery.​

On the morning of postoperative day one, the patient’s blood pressure was 133/65 mmHg, and magnesium sulfate was discontinued 24 hours following delivery. However, labetalol 200 mg two times a day was continued as blood pressures ranged from 114-155/55-86 bpm throughout the day. At this point, she had received a total amount of 3,340 mL intravenous fluids, which were also discontinued at this time. Her intake and output of fluids are shown in Table 4. The patient reported dyspnea with nasal congestion and coughing that improved with sitting up. Inspiratory stridor was heard, but the lungs were clear to auscultation. She had 1+ pitting edema of the lower extremities. On 4 L/min of supplemental oxygen via nasal cannula, oxygen saturations were 89-90%. By the late afternoon, her dyspnea was worsening, and she was requiring up to 10 L/min of supplemental oxygen. At this time, wet lung sounds were heard on auscultation. Labs showed increased leukocytosis but she remained afebrile (Table 2). Testing for COVID-19, influenza, and respiratory syncytial virus were negative. A computed tomography angiography (CTA), as shown in Figure 1, was ordered and showed pulmonary edema with possible pneumonia and pulmonary hypertension. Intravenous furosemide was started, and her urine output was monitored. Other interventions to improve breathing included pulmonary hygiene and the use of an incentive spirometer.

**Table 4:** Fluid intake and output POD, Post-operative day

​	Admission/POD 0​	POD 1​	POD 2​	POD 3​
Total Intake (mL)​	6930​	1400​	2844​	600​
Total Output (mL)​	7435​	3000​	3150​	2500​
Total Net (mL)​	-505​	-1600​	-306​	-1900​

On postoperative day two, the patient reported feeling better, but she was wheezing. Since the administration of furosemide, she had been voiding about every hour, amounting to about 3 L of urine output (Table 4). Blood pressures ranged from 120-200/63-106 bpm. Her oxygen requirements decreased to 3 L/min of oxygen, with her oxygen saturation at 93%. A hospitalist was consulted for further evaluation and management of her shortness of breath and respiratory failure. Harsh breath sounds were heard along with diffuse crackles on auscultation. The patient received nebulizer treatment as needed. The goal was to continue weaning oxygen as tolerated. She remained afebrile but her leukocyte count increased. Since her CTA was also suspicious for pneumonia, ceftriaxone and azithromycin were intravenously administered. Urinary streptococcus pneumonia and legionella antigens were negative. An echocardiogram confirmed the CTA findings of pulmonary hypertension, with a pulmonary artery pressure of 50 mmHg. Systolic function was preserved with a left ventricular ejection fraction greater than 67%.

On a post-operative day three, her oxygen saturation improved, while she was without supplemental oxygen. Despite being at high altitude, a pulse oximeter recorded saturations in the low 90s during rest, which only slightly decreased to 87% while walking. Blood pressures ranged from 123-180/65-95 bpm. She was discharged from the hospital later in the afternoon with the following oral medications: cefdinir, furosemide, and labetalol due to fluctuating blood pressure. She was advised to follow up with a pulmonologist or primary care provider in regard to her pulmonary complications and her obstetrician for routine postpartum care. Our patient’s pathology report of the placenta was not available until after she was already discharged. The pathologist noted a blood clot described as ragged and irregularly-shaped with a dusky red-brown color. It weighed 20 g and its greatest dimension was 6.0 cm. The cross-section of a fragment appeared laminated and well-organized. The report concluded that there was minimal to no evidence of abruption.

## Discussion

This case demonstrates several of the complications associated with preeclampsia and displays the necessity of rapid diagnosis and effective treatment. Our patient met the criteria for preeclampsia at 35 weeks when she presented to the clinic with consistently elevated blood pressure and proteinuria. The patient was appropriately scheduled for an induction of labor at 37 weeks. As a patient with preeclampsia approaches term, the placenta experiences further ischemia and there is an increased risk of associated complications and severity of presentation [7]. In fact, our patient’s condition progressed to preeclampsia with severe features at 37 weeks with an SBP greater than 160 mmHg during her induction of labor.

During labor, there was also a high suspicion of placental abruption. While the placenta can be sent to pathology, histological diagnosis of placental abruption is quite poor, with low sensitivity [8]. Our patient’s placenta, as examined by pathology, lacked features that suggested a placental abruption; however, our clinical findings seemed to indicate otherwise. The patient had significant vaginal bleeding upon artificial rupture of membranes. Furthermore, hysterotomy during the cesarean section revealed an excessive amount of blood in the uterine cavity. The placenta was easily removed from the womb as if the placenta had already detached from the uterine wall. Thus, the occurrence of a placental abruption based on clinical findings remains highly plausible.

The event of a placental abruption in a patient with preeclampsia is unsurprising as both conditions are attributed to similar risk factors [9]. This may be explained by a shared pathophysiologic mechanism of placental ischemia. Besides the patient’s diagnosis of preeclampsia, her risk for placental abruption was most likely compounded by her history of three abortions, two of which were induced but one was spontaneous. Evidence shows that the odds of placental abruption are greater in women with a history of spontaneous abortions compared to women without this history [10]. Diagnosing placental abruption can be challenging as specific, standardized criteria for diagnosis do not exist. Ultrasound findings are highly specific but not sensitive to placental abruption [11]. Furthermore, patients can be minimally symptomatic or asymptomatic, especially in the case of concealed placental abruptions. While placental abruption is relatively uncommon, affecting <1% of pregnancies, a patient who has multiple risk factors such as ours, should prompt providers to be on high alert for placental abruption [3]. Failure to identify and manage this obstetric complication can lead to adverse outcomes as placental abruption has been associated with increased rates of cardiac and pulmonary complications and even coma [3].

Pulmonary edema is defined as a collection of fluid within the alveolar spaces, which inhibits normal gas exchange. Our patient had several risk factors for the development of pulmonary edema, including her advanced maternal age, cesarean delivery, infection, and preeclampsia [1,4]. In the peripartum setting, pulmonary edema is postulated to be due to several physiologic and iatrogenic components [1,4]. Pregnancy alone results in a physiologic increase in circulating blood volume and pressure changes favoring extravascular fluid accumulation. In the setting of preeclampsia, increased vascular resistance and circulating angiogenic markers exacerbate pre-existing endothelial damage. This results in diffuse vascular fluid leakage, evidenced in our patient by severe range blood pressures and an elevated urine protein/creatinine ratio.

With the onset of severe range blood pressures in the setting of preeclampsia, the patient was treated with magnesium sulfate and labetalol prior to delivery. Studies have noted a relationship between the use of magnesium sulfate and the occurrence of pulmonary edema, possibly due to a negative inotropic effect or reduction in colloid osmotic pressure; however, the cause is still unclear [5]. Cardiac dysfunction is another postulated risk factor for the development of pulmonary edema. Our patient had no known pre-existing cardiac conditions. The echocardiogram demonstrated an enlarged left ventricle with preserved ejection fraction and severe pulmonary artery hypertension. We attribute the development of pulmonary artery hypertension to the additional resistance caused by her pulmonary edema. The ventricular findings are not concerning for acute cardiac dysfunction, as pregnancy alone can result in a physiologic increase in left ventricular muscle mass [12,13]. 

Furthermore, the influence of iatrogenic fluid administration in the development of pulmonary edema should not be overlooked. In the setting of preeclampsia with severe features, fluid administration is standard practice with the goal of preserving perfusion and reducing the risk of end-organ damage. Our patient received a total of 3,340 mL of intravenous fluids in the first 24 hours following admission. Fluid-restrictive policies in the setting of preeclampsia have been suggested by some authors with recommendations to limit the total amount of fluid administration to less than 2,000 mL and/or limit the rate of fluid administration to a maximum of 80 mL/hour [5,14]. Although our patient reportedly had a net fluid loss prior to the diagnosis of pulmonary edema, she had significant diuresis following the administration of furosemide. Further, iatrogenic fluid administration is a significant risk factor for the development of pulmonary edema throughout the peripartum period. In the postpartum time, the relationship between preeclampsia and pulmonary edema is often linked to iatrogenic fluid administration and physiological changes following delivery, resulting in large fluid shifts and decreased colloid osmotic pressure [4,5,15]. The development of pulmonary edema in the antepartum period can also be attributed to intravenous fluid administration associated with tocolytic use [4,15]. Additionally, research highlights an increased risk of acute pulmonary edema due to severe preeclampsia in patients of advanced maternal age, emphasizing the need for careful monitoring and management of fluid administration in these cases [14]. As research on this topic continues, providers must remain vigilant about the specific risk factors for each patient and monitor accordingly.

We encourage medical staff to urgently and aggressively address a complaint of new onset or worsening shortness of breath, as well as increasing oxygen demand in the peripartum time period. Although the occurrence of pulmonary edema is reported to be approximately 0.08%, the morbidity associated with the diagnosis is the leading cause of maternal mortality [4,5]. It is crucial for patients, families, and medical staff to be vigilant and well-informed about identifying signs and symptoms of pulmonary edema in the peripartum period, as this condition can be exceptionally severe.

## Conclusions

While preeclampsia is a common complication in pregnancy, the disease has systemic effects that compromise the health and safety of the mother and fetus. This case highlighted the presence of both placental abruption and pulmonary edema in a single patient. It further delved into various risk factors that could have contributed to the development of the disease. Ensuring medical personnel are aware of signs and symptoms is critical for prompt diagnosis and proper treatment. Given that preeclampsia can have a widespread impact, it is also important to involve multiple personnel in managing the patient and baby. With protocols in place and a strong, effective team, adverse events can be averted.
